# Anxiety and depression among Moroccan radiographers working in radiotherapy

**DOI:** 10.1539/eohp.2026-0015

**Published:** 2026-08-07

**Authors:** Jaouad Elkhalladi, Fatima Bouchareb, Fatima Zahra Aboukde, Fatima Zahra Outalmit, Jamal Tikouk, Abdelaaziz Bounabe, Mariam Akorzi, Mohamed El Fahssi, Hanane El Ghazouani, Ahmed Ouaamr, Mohammed Bouchekourte

**Affiliations:** 1 Faculty of Educational Sciences, Mohammed V University, Rabat, Morocco; 2 Faculty of Medicine and Pharmacy, Mohammed V University, Rabat, Morocco; 3 Higher Institute for Nursing Professions and Health Techniques, Agadir, Morocco; 4 Faculty of Law Economics and Social Sciences, Hassan II University, Casablanca, Morocco; 5 Faculty of Medicine and Pharmacy, Hassan II University, Casablanca, Morocco

**Keywords:** anxiety, depression, occupational health, radiotherapy

## Abstract

**Objectives:**

Radiographers working in specialized cancer treatment services are at high risk of psychiatric disorders because of the nature of their work and the types of patients receiving treatment there. The purpose of this study was to evaluate the prevalence of anxiety and depression among radiographers working in radiotherapy centers.

**Methods:**

The was a cross-sectional quantitative study of 55 radiographers working in four radiotherapy centers in Morocco between February and March 2023.

**Results:**

26% of radiographers had anxiety and 40% had depression. According to the findings, the factors that contribute the most to the emergence of anxiety are associated with sex, place of residence, physical activity, interpersonal relationships, and regret in choice of profession; for depression, the only factor found was radioprotection.

**Conclusions:**

This study revealed anxiety and depression among radiographers working in radiotherapy centers. Further studies are recommended to investigate causality and to examine additional factors that may cause anxiety and depression.

## Introduction

Anxiety and depression are among the most common psychiatric disorders and are often accompanied by significant comorbidities^1)^. They are strongly associated with numerous negative consequences for people’s well-being, quality of life, and even social relationships^2)^. Anxiety is strongly linked to occupational stress and working conditions, especially conflict and overload^3)^. Recent evidence suggests that depression may be influenced by adverse working conditions and the work environment, and exhibits several overlapping symptoms with burnout^4)^.

Health care is one of the most stressful fields in the world because of its high demands, high workload, and risk of illness and infection^5)^. Psychological stress is higher among nurses, especially those employed in oncology wards, according to research. Nursing, especially in cancer, is complex and challenging because patients undergo extensive and prolonged therapy, surgery, mental anguish and worry, despair, panic attacks, and even death^6)^. Furthermore, the high death rate, anxiety associated with cancer, and rising cancer patient population put those who care for these patients at risk of psychological distress, such as anxiety, sadness, and especially burnout^7)^.

Among healthcare professionals in this field is the radiographer, who performs radiology examinations^8)^ and is involved in the diagnosis and treatment of cancer. In the radiotherapy department, the radiographer is a crucial member of the radiotherapy team, alongside the radiation oncologist and the medical physicist. They participate in every step of the process, including patient preparation, positioning, dosimetry, and treatment. They also take part in the training of future radiographers and oversee pharmacy and radioactive element management^9)^. Furthermore, a radiographer is crucial to the assessment, management, and application of preventive measures^10)^. It is not unusual for radiographers, among healthcare workers, to experience high levels of stress and burnout^11)^. Because radiographers in radiotherapy work closely with cancer patients and their caregivers, physical and psychological stress is often caused by patient characteristics, recurrent fatalities, and other stressors related to the workload and organization of oncology personnel.

In the United Kingdom, the prevalence of emotional exhaustion among radiographers was 38%^12)^. Furthermore, research conducted in Italy during the COVID-19 pandemic revealed that the pandemic significantly affected personal fulfillment, but not depersonalization or emotional exhaustion. This may be because, even in the absence of the pandemic, radiographers already exhibited high levels of burnout and therefore remained above the cutoff scores for emotional exhaustion and depersonalization^13)^.

Given the paucity of research on this topic among radiographers in radiotherapy, and the importance of healthcare professionals’ well-being for the quality of care, patient management, and the improvement of the profession, practice, and psychological support, we conducted this study to assess the level of work-related anxiety and depression among radiographers in the radiotherapy department, along with the various factors contributing to these psychological disorders.

## Methods

### Study type

This descriptive cross-sectional study assessed levels of anxiety and depression among radiographers in radiotherapy centers.

### Study environment

The survey was performed at four radiotherapy centers in Rabat, Agadir, Casablanca, and Marrakech. These locations were chosen based on the presence of radiotherapy centers, the number of radiographers working in these centers, and the ease of access to information.

### Population and sample

Our study population consisted of 64 radiographers practicing in four radiotherapy centers. In the Moroccan context, the term “radiographer” may refer to professionals working in both diagnostic imaging and radiotherapy. However, this study was conducted exclusively in radiotherapy centers; therefore, all included radiographers were involved in radiotherapy procedures (treatment delivery) and did not perform diagnostic imaging activities. Only 55 (n=55) radiographers responded to our data collection questionnaire, with an overall response percentage of 85.93% distributed as follows: UHC (university hospital center) of Rabat (87.50%), CHU of Casablanca (79.16%), CHU of Marrakech (87.50%), and CHU of Agadir (100%). The four radiotherapy centers were selected based on both practical and scientific considerations. Agadir was included due to its accessibility and proximity to the research team, which facilitated data collection. In addition, three well-established centers in Morocco, Rabat, Casablanca, and Marrakech, were selected to better reflect variations in workload and professional experience. Newer centers such as Fez, Tangier, and Oujda were not included, as they currently have lower activity levels and fewer radiographers.

A post hoc power analysis was conducted using G*Power (version 3.1). Assuming a moderate effect size (Cohen’s d=0.5) and a significance level of 0.05, the achieved power for a sample of 55 participants was estimated at 0.73. This indicates an acceptable level of statistical power for detecting moderate effects.

### Data collection tool

An online questionnaire comprising three sections was developed with reference to the literature. The first part covers socio-demographic data, lifestyle, and working conditions. The second part involves the HADS (Hospital Anxiety and Depression Scale), which measures depression and anxiety in participants. The latter is a self-assessment scale and a reliable instrument developed by Zigmond and Snaith^14)^.

The HADS was chosen because it is easy to understand, quick to apply, and comprises few items. The HADS, which addresses the variables of interest (anxiety and depression), has demonstrated good psychometric characteristics in people with various types of illness and is applicable in various contexts^15)^. The scale contains 14 multiple-choice questions, with two subscales: anxiety (HAD-A) and depression (HAD-D), with seven items in each domain. Scores for each item range from zero to three, and each subscale’s total score falls between 0 and 21. To interpret the scores of the two subscales, we consider that the higher the score, the more likely the person is to develop anxiety and/or depressive disorder^14)^. To identify anxious and depressive symptoms, the following interpretation can be proposed for each of the scores (A and D): 7 or less: no symptoms; between 8 and 10: borderline symptoms; 11 and over: definite symptoms. For the purpose of this study, only scores ≥11 were considered to define the presence of anxiety and depression, while borderline scores (8–10) were not included in the prevalence estimates. The version used is the French version^16)^.

In addition, radiation protection was assessed using a self-reported item asking participants to rate the overall quality of radiation protection in their workplace (Excellent/Good/Average/Poor), taking into account aspects such as availability of protective equipment and adherence to safety procedures. This measure reflects participants’ perceptions rather than an objective assessment of compliance, as no dosimetric or observational data were collected.

### Questionnaire reliability and validity

Before the main data collection, a pre-test of the questionnaire was conducted with two radiology teachers and two radiographers from the radiotherapy department who were not part of the study sample. This step helped ensure that the questions were clear, well-structured, and easy to understand. The internal consistency of the questionnaire was then assessed using Cronbach’s alpha based on the responses from the main study sample. The obtained value (0.754) indicates an acceptable level of reliability.

### Data analysis

The data were analyzed using IBM SPSS version 25 software. The chi-square test was used to compare two independent qualitative variables and detect significant differences. A p-value ≤ 0.05 was considered statistically significant.

## Results

According to Table 1, 63.6% of the participants were female, 72.7% were under 35 years old, 94.5% lived in urban areas, 54.5% were married, 76.4% had a bachelor’s degree, and 61.8% had a monthly income between 6,000 and 7,000 Moroccan dirhams (approximately US＄650–760).

**Table 1.** Associations of anxiety and depression with socio-demographic characteristics and lifestyle

Variable		N (%)	Anxiety				Depression			
			No	Uncertain	Yes	*p*-value	No	Uncertain	Yes	*p*-value
**Sex**						.020*				.544
	Female	35 (63.6)	9	17	9	V=.378	9	11	15	V=.149
	Male	20 (36.4)	12	3	5		8	5	7	
**Age (years)**						.197				.257
	≤ 35	40 (72.7)	15	17	8	V=.243	12	14	14	V=.222
	> 35	15 (27.3)	6	3	6		5	2	8	
**Residence**						.010*				.457
	Urban	52 (94.5)	21	20	11	V=.411	17	15	20	V=169
	Suburban	3 (5.5)	0	0	3		0	1	2	
**Family situation**						.877				.268
	Single	25 (45.5)	9	10	6	V=.069	5	8	12	V=219
	Married	30 (54.5)	12	10	8		12	8	10	
**Education level**						.845				.987
	Bachelor	42 (76.4)	16	16	10	V=.078	13	12	17	V=.022
	Master	13 (23.6)	5	4	4		4	4	5	
**Monthly income**						.689				.085
	< 7,000 Dirhams	34 (61.8)	11	13	10	V=.143	8	12	14	V=.273
	7,000–8,000 Dirhams	18 (32.7)	8	6	4		6	4	8	
	> 8,000 Dirhams	3 (5.5)	2	1	0		3	0	0	
**Number of hours of sleep/night**						.187				.178
	< 5 hours	5 (9.1)	2	0	3	V=.237		0	4	V=.239
	5-8 hours	49 (89.1)	19	19	11		16	15	18	
	> 8 hours	1 (1.8)	0	1	0		0	1	0	
**Balanced diet**						.228				.162
	No	10 (18.2)	2	5	3	V=.226	1	3	6	V=.244
	Yes	7 (12.7)	5	2	0		1	4	2	
	More or less	38 (69.1)	14	13	11		15	9	14	
**Physical activity**						.050*				.136
	No	43 (78.2)	16	13	0	V=.330	11	12	20	V=.269
	Yes	12 (21.8)	5	7	14		6	4	2	
**State of stress**						.192				.085
	No	31 (56.4)	15	10	6	V=.245	13	9	9	V=.299
	Yes	24 (43.6)	6	10	8		4	7	13	

*p ≤ .05; Cramer’s V indicates effect size (0.10=small, 0.30=medium, 0.50=large).

The results of Table 1 also show that 89.1% of participants slept 5-8 hours per day, 69.1% reported a balanced diet, 78.2% did not engage in physical activity, and 43.6% reported stress.

Additionally, the results showed statistically significant relationships between anxiety and sex (p=0.02), residence (p=0.01), and physical activity (p=0.05). However, no significant relationship was found with age, family situation, level of education, monthly income, hours of sleep, balanced nutrition, and stress status. Regarding depression, no statistically significant associations were observed with socio-demographic characteristics or lifestyle factors.

As shown in Table 2, the participants suffered from workload (83.6%), poor organization (72.7%), and problematic interpersonal relationships (34.5%). In addition, only 7.3% of participants rated the quality of radiation protection as excellent, only 34.5% had a seniority of more than 10 years, and 50.9% regretted their choice of radiology as a career.

**Table 2.** Associations of anxiety and depression with working conditions

Variables		N (%)	Anxiety				Depression			
			No	Uncertain	Yes	*p*-value	No	Uncertain	Yes	*p*-value
**Workload**						.556				.353
	No	9 (16.4)	2	4	3	V=.146	1	3	5	V=.195
	Yes	46 (83.6)	19	16	11		16	13	17	
**Poor organization**						.229				.663
	No	15 (27.3)	5	8	2	V=.232	6	4	5	V=.122
	Yes	40 (72.7)	16	12	12		11	12	17	
**Interpersonal relationships**						.042*				.787
	No	36 (65.5)	10	17	9	V=.340	10	11	15	V=.093
	Yes	19 (34.5)	11	3	5		7	5	7	
**Radiation protection**						.209				.017*
	Excellent	4 (7.3)	3	0	1	V=.277	3	1	0	V=.374
	Good	22 (40.0)	9	9	4		10	7	5	
	Average	14 (25.4)	6	6	2		4	3	7	
	Poor	15 (27.3)	3	5	7		0	5	10	
**Seniority (years)**						.504				.090
	< 5	17 (30.9)	7	8	2	V=.174	3	7	7	V=.270
	5-10	19 (34.6)	6	6	7		4	7	8	
	> 10	19 (34.5)	8	6	5		10	2	7	
**Regret about career choice**						.041*				.100
	No	27 (49.1)	11	13	3	V=.341	11	9	7	V=.290
	Yes	28 (50.9)	10	7	11		6	7	15	

*p ≤ .05; Cramer’s V indicates effect size (0.10=small, 0.30=medium, 0.50=large).

Moreover, the results showed statistically significant relationships between anxiety and interpersonal relationships (p=0.042), and regret about career choice (p=0.041). However, no significant relationship was found with workload, poor organization, radiation protection, and seniority. Concerning depression, a single significant relationship was found, namely with radiation protection (p=0.017). However, no significant relationship was found with workload, poor organization, interpersonal relationship, regret about career choice, and seniority.

**Fig. 1.**
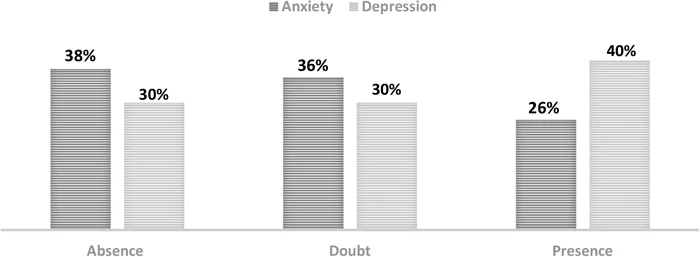
The prevalence of anxiety and depression among radiographers in radiotherapy

Figure 1 shows that 26% of participants had anxiety and 40% had depression, based on HADS scores ≥11, indicating definite symptoms. Borderline cases (scores 8–10) were not included in these prevalence estimates.

## Discussion

### Anxiety

The results obtained show that the prevalence of anxiety among radiographers is 26%, with a score of 8.65. This is in line with numerous studies conducted on oncology staff and healthcare personnel in general. Indeed, a 2018 Brazilian study on doctors working in cancer treatment hospitals revealed that 44 of 227 doctors experienced anxiety, as assessed with the same scale (HADS)^17)^. Furthermore, another study carried out in Ukraine indicates that 48.2% of nurses working in psychiatric hospitals had anxiety symptoms, with the associated factors being age, family situation, and level of education^18)^. However, our findings indicate no association between anxiety and these factors. On the other hand, the results of the present study showed a significant association between sex and anxiety (p=0.020). Female sex was a risk factor for the occurrence of moderate to severe anxiety symptoms^19)^. Also, a Greek study conducted by Tselebis (2006) on 76 nurses and 66 doctors reported that nurses had the lowest anxiety scores, while female doctors had the highest, followed by male nurses^20)^.

Numerous studies have identified work-related stress as a risk factor for anxiety. An Italian study conducted in 2022, examining professional burnout among radiographers, found that, as a group of professionals, radiographers had high levels of the first two stages of professional burnout prior to the pandemic, indicating that stress is the primary cause of burnout^13)^. However, according to our study, stress affects 43.6% of radiographers, but there is no association between stress and the onset of anxiety (p=0.19), contrary to the literature.

In addition, there was a significant association between regret at having chosen this profession and anxiety (p=0.041). Indeed, regret regarding the profession may be associated with lower motivation and higher perceived occupational stress, which could contribute to anxiety symptoms. In an American study, 7.6% of participating radiologists expressed a wish to leave their institution^21)^. Furthermore, there was a significant relationship between anxiety and interpersonal relationships (p=0.042), as well as anxiety and physical activity (p=0.05). In the same sense, physical activity has been associated with lower levels of anxiety and depressive symptoms in previous studies^22)^. Previous research has also suggested that regular physical activity may be associated with improved psychological well-being and lower anxiety levels^23,24)^.

The results of our study also showed a significant association between place of residence and anxiety (p=0.01), with the majority of participants (94.5%) living in urban areas. Urban environments may expose individuals to different stressors that could be related to anxiety symptoms. This finding is partly consistent with a study conducted in Kazakhstan among radiology professionals, which also reported an association between place of residence and anxiety (p=0.001), with rural professionals more likely to report anxiety-related concerns^25)^. This difference may be explained by contextual variations, particularly between study conditions and real-world working conditions, as well as differences in work organization, access to resources, and the professional environment.

### Depression

Analysis of the collected data revealed a prevalence of depression in 40% of radiographers, with a score of 9.3 on the HADS scale. These results are in line with data from a Ukrainian study that found 52% of nurses to be depressed^18)^. Moreover, according to a Brazilian study of oncologists, 12.3% had depression, mainly attributed to stress and job dissatisfaction^17)^.

Furthermore, there was no significant relationship between depression and physical activities (p=0.136). This is inconsistent with a systematic review by Mammen and Faulkner (2013), in which 25 of the 30 studies reviewed reported an inverse association between baseline physical activity and the subsequent risk of depression^26)^. In addition, Swedish research demonstrated that physically inactive older adults exhibited higher levels of depressive symptoms than those who regularly engaged in light or strenuous physical activity^27)^. Indeed, physical activity has a positive effect on well-being and depression reduction for healthcare professionals^28)^, patients^29)^, and students in the healthcare field^30)^.

Moreover, low income appears to be a significant factor in mental health. A study examining the relationship between depression and sociodemographic traits, particularly salary, revealed that the frequency of depression was associated with income^31)^. However, our study revealed no significant relationship between monthly income and depression (p=0.08). This result is in line with another study carried out on teachers in Brazil, which revealed the absence of a significant relationship between income and the prevalence of psychiatric disorders^32)^.

Additionally, our results showed a significant association between the perceived level of radiation protection and depression (p=0.017). However, this variable reflects subjective perceptions rather than objective radiation exposure, since no dosimetric measurements were available. Consequently, the observed association likely reflects psychological mechanisms related to perceived occupational risk and workplace safety concerns. Previous studies have shown that healthcare professionals working in radiology settings are frequently exposed to elevated levels of stress and psychological burden, which may increase vulnerability to burnout, anxiety, and depressive symptoms^33)^.

The results of our study also showed the absence of a significant relationship between workload and anxiety and depression, even though 83.6% of participants in our study reported the presence of workload. This is at odds with a set of studies that have shown the presence of a significant relationship between these variables. In the same vein, a Chinese study of 3,666 radiologists showed a significant relationship between workload and depression^34)^. Another American study of 228 radiologists revealed a significant relationship between workload and burnout^35)^.

Additionally, the prevalence of anxiety and depression in our study was slightly lower than that other studies of radiographers. In the same context, an American study of 173 radiographers showed that the largest proportion of radiographers had mild symptoms of depression (35.8%) and anxiety (32.9%)^36)^. Another Iranian study of 281 healthcare professionals revealed that radiographers were the most likely to feel anxiety and stress, compared to doctors and other categories^37)^. Indeed, radiographers in radiotherapy, responsible for the daily care of patients, are confronted throughout their day with obstacles that hinder adherence to the planned plan, causing stress and sometimes demotivation in the work, especially when the radiographer is forced to prioritize patients^38)^.

### Limitations

Although this multicenter study included four of the seven radiotherapy centers in Morocco, caution should be exercised when generalizing the findings to all radiographers working in radiotherapy nationwide. Notably, this is the first study to specifically investigate anxiety and depression among radiographers working in radiotherapy in Morocco. However, several limitations should be considered when interpreting the findings of this study. First, the relatively small sample size may limit the generalizability of the results. Although a post hoc power analysis conducted using G*Power indicated an acceptable level of statistical power (1–β=0.73), the study may still have been underpowered to detect smaller effect sizes. In addition, multiple bivariate chi-square analyses were performed without adjustment for multiple comparisons, which may have increased the risk of Type I error. For this reason, the findings should be interpreted with caution and viewed as exploratory rather than confirmatory. While effect sizes (Cramer’s V) were reported to provide more information about the strength of associations, most were small, suggesting limited practical significance.

Furthermore, some relevant factors that may influence anxiety and depression were not included in this study, and no adjustment for potential confounders was performed. In addition, radiation protection was assessed using a self-reported measure based on participants’ perceptions, without objective verification (e.g., dosimetric data), which may introduce perception bias and limit the accuracy of this variable. Given the relatively small sample size, multivariable analyses were not undertaken in order to avoid model overfitting. Finally, the cross-sectional design does not allow for conclusions about causality. In addition, the limited number of studies focusing specifically on radiographers made it challenging to fully compare and contextualize the findings. Moreover, the reliance on studies conducted among other healthcare professionals for contextual comparison may limit the direct applicability of some interpretations, given differences in roles, training, and occupational exposure.

## Conclusions

In conclusion, this study highlights the presence of anxiety and depressive symptoms among radiographers and identifies several occupational, individual, and sociodemographic factors that may be associated with these outcomes. These findings emphasize the need to better consider occupational factors in order to support the mental well-being of this population. Given the exploratory nature of the study, further research with larger samples and more robust designs is needed to confirm these results and deepen understanding of the observed associations. Future studies could explore potential psychosocial interventions that may support the mental well-being of radiographers.

## References

[1] Kalin NH. The critical relationship between anxiety and depression. *Am J Psychiatry*. 2020; 177(5): 365-367. 10.1176/appi.ajp.2020.20030305 32354270

[2] Hanna M, Strober LB. Anxiety and depression in Multiple Sclerosis (MS): Antecedents, consequences, and differential impact on well-being and quality of life. *Mult Scler Relat Disord*. 2020; 44:102261. 10.1016/j.msard.2020.102261 PMC7719086 32585615

[3] D’emeh WM, Yacoub MI, Shahwan BS. Work-related stress and anxiety among frontline nurses during the COVID-19 pandemic: A cross-sectional study. *J Psychosoc Nurs Ment Health Serv*. 2021; 59(8): 31-42. 10.3928/02793695-20210322-02 34110949

[4] Bakker AB, Costa PL. Chronic job burnout and daily functioning: A theoretical analysis. *Burn Res*. 2014; 1(3): 112-119.

[5] Hummel S, Oetjen N, Du J, et al. Mental health among medical professionals during the COVID-19 pandemic in eight European countries: Cross-sectional survey study. *J Med Internet Res*. 2021; 23(1):e24983. 10.2196/24983 PMC7817254 33411670

[6] Ploukou S, Panagopoulou E. Playing music improves well-being of oncology nurses. *Appl Nurs Res*. 2018; 39: 77-80. 10.1016/j.apnr.2017.11.007 29422181

[7] Medisauskaite A, Kamau C. Prevalence of oncologists in distress: Systematic review and meta-analysis. *Psychooncology*. 2017; 26(11): 1732-1740. 10.1002/pon.4382 28116833

[8] Elkhalladi J, Sefrioui A. The impact of the flipped classroom on the development of radiology students’ soft skills. *J Radiol Nurs*. 2024; 43(2): 142-146.

[9] Boisbouvier S, Tachin C, Lecoeur A, Le Tallec P. [The evolving career profile of radiation therapists]. *Cancer Radiother*. 2020; 24(6-7): 730-735. 10.1016/j.canrad.2020.05.009 32807686

[10] Bai X. Application of the PDCA cycle for nursing safety management in radiology department. *J Radiol Nurs*. 2023; 42(2): 241-244.

[11] Diggens J, Chesson T. Do factors of emotion-focussed patient care and communication impact job stress, satisfaction and burnout in radiation therapists? *J Radiother Pract*. 2014; 13(1): 4-17.

[12] Probst H, Griffiths S, Adams R, Hill C. Burnout in therapy radiographers in the UK. *Br J Radiol*. 2012; 85(1017): e760-e765. 10.1259/bjr/16840236 PMC3487097 22253352

[13] Zanardo M, Cornacchione P, Marconi E, et al. Occupational burnout among radiation therapy technologists in Italy before and during COVID-19 pandemic. *J Med Imaging Radiat Sci*. 2022; 53(1): 58-64. 10.1016/j.jmir.2021.12.004 PMC8763333 35115275

[14] Zigmond AS, Snaith RP. The hospital anxiety and depression scale. *Acta Psychiatr Scand*. 1983; 67(6): 361-370. 10.1111/j.1600-0447.1983.tb09716.x 6880820

[15] Schmidt DRC, Dantas RAS, Marziale MHP. [Anxiety and depression among nursing professionals who work in surgical units]. *Rev Esc Enferm USP*. 2011; 45(2): 487-493. 10.1590/s0080-62342011000200026 21655802

[16] Untas A, Aguirrezabal M, Chauveau P, Leguen E, Combe C, Rascle N. Anxiété et dépression en hémodialyse: Validation de l’Hospital Anxiety and Depression Scale (HADS). *Nephrol Ther*. 2009; 5(3): 193-200. 10.1016/j.nephro.2009.01.007 19346177

[17] Paiva CE, Martins BP, Paiva BSR. Doctor, are you healthy? A cross-sectional investigation of oncologist burnout, depression, and anxiety and an investigation of their associated factors. *BMC Cancer*. 2018; 18(1): 1044. 10.1186/s12885-018-4964-7 PMC6203972 30367614

[18] Tsaras K, Papathanasiou IV, Vus V, et al. Predicting factors of depression and anxiety in mental health nurses: A quantitative cross-sectional study. *Med Arch*. 2018; 72(1): 62-67. 10.5455/medarh.2017.72.62-67 PMC5789556 29416221

[19] Huang CLC, Wu MP, Ho CH, Wang JJ. Risks of treated anxiety, depression, and insomnia among nurses: A nationwide longitudinal cohort study. *PLoS One*. 2018; 13(9):e0204224. 10.1371/journal.pone.0204224 PMC6155527 30252873

[20] Tselebis A, Gournas G, Tzitzanidou G, Panagiotou A, Ilias I. Anxiety and depression in Greek nursing and medical personnel. *Psychol Rep*. 2006; 99(1): 93-96. 10.2466/pr0.99.1.93-96 17037454

[21] Higgins MCSS, Siddiqui AA, Kosowsky T, et al. Burnout, professional fulfillment, intention to leave, and sleep-related impairment among radiology trainees across the United States (US): A multisite epidemiologic study. *Acad Radiol*. 2022; 29(suppl 5): S118-S125. 10.1016/j.acra.2022.01.022 35241358

[22] González Aguña A, Fernández Batalla M, Herrero Jaén S, Sierra Ortega A, Martínez Muñoz ML, Santamaría García JM. Active and healthy confinement: Care recommendations on activity, sleep and relationships. *Healthcare (Basel)*. 2023; 11(12): 12. 10.3390/healthcare11121773 PMC10298066 37372890

[23] Goodarzi S, Teymouri Athar MM, Beiky M, et al. Effect of physical activity for reducing anxiety symptoms in older adults: A meta-analysis of randomized controlled trials. *BMC Sports Sci Med Rehabil*. 2024; 16(1): 153. 10.1186/s13102-024-00947-w PMC11251295 39014515

[24] Oliveira J, Monteiro D, Jacinto M, et al. Physical activity, anxiety, depression, and body image in trans individuals: An exploratory study. *Healthcare (Basel)*. 2024; 12(10): 10. 10.3390/healthcare12101008 PMC11121412 38786418

[25] Darbayeva A, Dautov T, Zhakhina G, et al. Diagnostic radiology services and occupational radiation anxiety in Kazakhstan. *Int J Environ Res Public Health*. 2025; 22(12): 1785. 10.3390/ijerph22121785 PMC12732935 41464419

[26] Mammen G, Faulkner G. Physical activity and the prevention of depression: A systematic review of prospective studies. *Am J Prev Med*. 2013; 45(5): 649-657. 10.1016/j.amepre.2013.08.001 24139780

[27] Lindwall M, Rennemark M, Halling A, Berglund J, Hassmén P. Depression and Exercise in Elderly Men and Women: Findings from the Swedish National Study on Aging and Care. Published online January 1, 2007. 10.1123/japa.15.1.41 17387228

[28] Hiestand S, Waage S, Forthun I, Pallesen S, Bjorvatn B. Factors leading to excessive fatigue in nurses - a three-year follow-up study. *BMC Nurs*. 2024; 23(1): 446. 10.1186/s12912-024-02066-w PMC11218166 38951772

[29] Kaur S, Cherukuri SHS, Murshed SM, Purev-Ochir A, Abdelmassih E, Hanna F. The impact of regular physical activity on the mental health and well-being of dementia patients in high-income countries-a systematic scoping review. *Geriatrics (Basel)*. 2024; 9(4): 98. 10.3390/geriatrics9040098 PMC11353956 39195128

[30] Nguyen MH, Do TX, Nguyen TT, et al. Fear of COVID-19, healthy eating behaviors, and health-related behavior changes as associated with anxiety and depression among medical students: An online survey. *Front Nutr*. 2022; 9:938769. 10.3389/fnut.2022.938769 PMC9538633 36211498

[31] Akhtar-Danesh N, Landeen J. Relation between depression and sociodemographic factors. *Int J Ment Health Syst*. 2007; 1(1): 4. 10.1186/1752-4458-1-4 PMC2241832 18271976

[32] dos Reis EJFB, Carvalho FM, de Araújo TM, Porto LA, Silvany Neto AM. [Work and psychological distress among public school teachers in Vitória da Conquista, Bahia State, Brazil]. *Cad Saude Publica*. 2005; 21(5): 1480-1490. 10.1590/s0102-311x2005000500021 16158154

[33] Amin SI, Mahdy RS, El-Shafei DA, et al. Burnout syndrome, anxiety, and depression symptoms among workers in radiation field. *Middle East Curr Psychiatry Ain Shams Univ*. 2024; 31(1): 66.

[34] Luo S, Zhang Y, Wang P, et al. The moderating role of resilience in the association between workload and depressive symptoms among radiology residents in China: Results from a nationwide cross-sectional study. *Eur Radiol*. 2024; 34(1): 695-704. 10.1007/s00330-023-10021-7 37566268

[35] Ganeshan D, Rosenkrantz AB, Bassett RL Jr, Williams L, Lenchik L, Yang W. Burnout in academic radiologists in the United States. *Acad Radiol*. 2020; 27(9): 1274-1281. 10.1016/j.acra.2019.12.029 32037261

[36] Sackett M, Clark KR, Webster TL. Shift Work and mental health among radiologic technologists. *Radiol Technol*. 2023; 94(3): 168-179. 36631221

[37] Gong H, Zhang SX, Nawaser K, et al. The mental health of healthcare staff working during the covid-19 crisis: Their working hours as a boundary condition. *J Multidiscip Healthc*. 2021; 14: 1073-1081. 10.2147/JMDH.S297503 PMC8122682 34007182

[38] Le Tallec P, Gesbert C, Mercier C, Crenn É. Compétence éthique du manipulateur en radiothérapie dans la gestion d’une crise. *Cancer Radiother*. 2022; 26(6-7): 841-845. 10.1016/j.canrad.2022.06.015 36075832

